# Long Pentraxin 3 as a Broader Biomarker for Multiple Risk Factors in End-Stage Renal Disease: Association with All-Cause Mortality

**DOI:** 10.1155/2019/3295725

**Published:** 2019-06-16

**Authors:** Maria João Valente, Susana Rocha, Susana Coimbra, Cristina Catarino, Petronila Rocha-Pereira, Elsa Bronze-da-Rocha, José Gerardo Oliveira, José Madureira, João Carlos Fernandes, Maria do Sameiro-Faria, Vasco Miranda, Luís Belo, Alice Santos-Silva

**Affiliations:** ^1^UCIBIO, REQUIMTE, Laboratório de Bioquímica, Faculdade de Farmácia da Universidade do Porto, Porto, Portugal; ^2^CESPU, Instituto de Investigação e Formação Avançada em Ciências e Tecnologias Saúde (IINFACTS), Gandra-Paredes, Portugal; ^3^Universidade da Beira Interior, Centro de Investigação em Ciências da Saúde, Covilhã, Portugal; ^4^Clínica de Hemodiálise do Porto, Porto, Portugal; ^5^Centro de Investigação em Tecnologias e Serviços de Saúde (CINTESIS), Faculdade de Medicina da Universidade do Porto, Portugal; ^6^NefroServe, Centro de Hemodiálise de Nossa Senhora Da Franqueira, Barcelos, Portugal; ^7^NefroServe, Clínica de Hemodiálise de Viana do Castelo, Viana do Castelo, Portugal; ^8^Unidade de Hemodiálise, Hospital Agostinho Ribeiro, Felgueiras, Felgueiras, Portugal; ^9^Clínica de Hemodiálise de Gondomar, Gondomar, Portugal

## Abstract

Persistent inflammation in end-stage renal disease (ESRD) patients is known to underlie the progression of chronic kidney disease and to be associated with multiple risk factors including malnutrition, atherosclerosis, and cardiovascular disease (CVD). The acute-phase protein pentraxin 3 (PTX3) has a proven potential as a local inflammatory biomarker, but its clinical utility in ESRD remains unclear. Circulating levels of PTX3 and classical inflammatory mediators, including the clinical prototypical C-reactive protein (CRP), were assessed in 246 ESRD patients on dialysis and analysed in relation to the lipid profile, adipokine levels, and nutritional, cardiac, and renal fibrosis markers. Occurrence of deaths was recorded for the following year. Contrarily to the classical inflammatory markers, PTX3 levels were negatively correlated with nutritional markers and associated with a less atherogenic lipid profile. Levels of the cardiac and renal fibrosis markers and of the oxidized LDL/LDL-C ratio were found to be independent determinants of PTX3 concentration. When comparing inflammatory mediators, the increase in the PTX3 levels was the only predictor of all-cause mortality in dialysis patients in a survival model adjusted to all markers under study, other than the inflammatory ones, besides common confounding factors in dialysis. Data support the clinical applicability of PTX3 as a broader inflammatory biomarker than the classical ones, presenting a close association with inflammation, malnutrition, CVD, and renal fibrosis and a great potential to predict all-cause mortality in dialysis patients. The pleiotropic character of PTX3 may be of clinical relevance, and it could be targeted to ameliorate the high morbidity and mortality associated with ESRD.

## 1. Introduction

Chronic inflammation has been implicated in the progression and outcome of chronic kidney disease (CKD) patients and is a distinctive condition in patients undergoing dialysis [1]. The prevalent state of inflammation in end-stage renal disease (ESRD) may result from a multiplicity of causes, including the dialysis procedure *per se*, reduced renal function, intercurrent clinical events, and the presence of comorbid conditions [2].

Several studies showed an association of systemic biomarkers of inflammation, such as C-reactive protein (CRP), interleukin-6 (IL-6), and tumor necrosis factor-alpha (TNF-*α*), with progression of CKD and with higher risk for morbidity and mortality, particularly due to cardiovascular (CV) events [3–6]. Moreover, inflammation itself appears to contribute to the progression of other common features in ESRD, such as atherosclerosis, protein-energy wasting, and heart-related complications [7–9]. Given the potential of inflammatory markers for early diagnosis, prognosis, and monitoring of CKD and their value as predictive markers of mortality risk and as potential therapeutic targets, an increased interest in new biomarkers of inflammation has emerged. Several novel inflammatory biomarkers have been reported in recent years, but CRP remains the prototypical clinical indicator of inflammation in patients undergoing haemodialysis [10], with a proven predictive potential for cardiovascular morbidity and mortality in this population [11].

Long pentraxin 3 (PTX3), one of the emerging biomarkers of inflammation, is a modulator of the immunoinflammatory response belonging to the pentraxin superfamily that includes CRP. Contrarily to CRP, PTX3 is produced at sites of inflammation, by resident and inflammatory cells, such as myeloid and epithelial cells, lymphatic and vascular endothelial cells, smooth muscle cells, and fibroblasts [12]; evidence shows that PTX3 is also expressed and released by adipose tissue, with a possible interaction of this modulator with both atherosclerosis and obesity [13, 14]; PTX3 does not respond to IL-6 stimulus, like CRP, but, instead, its expression is induced by lipoproteins, by the proinflammatory stimulus of TNF-*α*, and by the activation of Toll-like receptors [12, 15].

Several lines of investigation support PTX3 as a useful biomarker of inflammation under a wide range of clinical conditions, including acute myocardial infarction, atherosclerotic lesion, rheumatoid arthritis, and septic shock [16–19]. In CKD patients, higher PTX3 levels are associated with decreased renal function [20, 21] and appear to have a predictive value for mortality, independently of traditional risk factors [21–23].

Importantly, PTX3 has been described as a multifaceted modulator which appears to be able to bind to multiple ligands and exert a complexity of both protective and harmful effects in different clinical settings including inflammation, innate immunity, tissue repair, atherosclerosis, and CVD [24, 25]. Deciphering the contribution of the balance of the physiologic effects of this pentraxin to ESRD and associated comorbidities will enable the development of targeted therapeutic approaches in dialysis patients.

This study is aimed at evaluating the potential of PTX3 as a biomarker for multiple risk factors associated with ESRD, particularly inflammation, malnutrition, CVD, and renal fibrosis. The predictive value of PTX3 for all-cause mortality in prevalent dialysis patients was further compared to that of the classical inflammatory biomarkers, with special emphasis to the clinical standard CRP.

## 2. Materials and Methods

### 2.1. Subjects

All procedures performed in studies involving human participants were in accordance with the ethical standards of the Ethics Committee from the Faculty of Pharmacy, University of Porto, and the Directors of Dialysis Clinics involved in the study. Informed consent was obtained from all individual participants included in the study. According to the most recent data on the national prevalence of renal replacement therapy, by the end of 2018 a total of 12 227 ERSD patients were undergoing haemodialysis in Portugal, from which 4 901 were between the ages of 65 and 80 years old [26]. In order to include a representative sample of this population, 318 ESRD patients from 5 different Dialysis Clinics in the Northern region of Portugal were selected during the period from February to July 2017. From these, patients with known active infectious diseases, recent trauma or neoplastic diseases, or undergoing dialysis for less than 90 days were excluded from the study, resulting in a total of 246 ESRD patients kept under study. Dialysis treatment was performed using FX-class® high-flux polysulfone dialyzers (1.4–2.2 m^2^) (Fresenius, Germany); only 12.2% (*n* = 30) were under high-flux haemodialysis, while 87.8% of patients (*n* = 216) were under online haemodiafiltration. From March to April 2017, 44 volunteers without history of renal disease were selected for the control group. From these, 22 healthy volunteers were kept under study, based on normal haematological and biochemical data, while 22 subjects were excluded due to high total cholesterol levels, mild hypertension, anaemia, or therapy with drugs that could have influence on the parameters in the study, including antihypertensive and antidyslipidemic drugs. Subjects from the control group and ESRD patients presented similar distribution for gender, for body mass index (BMI), and, as far as possible, for age. Clinical data from ESRD patients were gathered at the Dialysis Clinics at the beginning of the study, and along the following year, a clinical follow-up was conducted to identify cases of death. A total of 26 deceased patients (10.6%) were reported over the one-year follow-up period, with miscellaneous causes of death, including cardiovascular causes, cachexia, infectious diseases, or others. Demographic data from both controls and patients, as well as CKD aetiology and dialysis-related data from the latter group, are presented in Table 1.

### 2.2. Sample Collection

Sample collection from ESRD patients took place immediately before a midweek dialysis therapy session. Blood samples from both controls and patients were collected into tubes with and without anticoagulant (ethylenediaminetetraacetic acid), in order to obtain plasma and serum, respectively, and processed within 2 hours. Aliquots were immediately stored at −80°C until assayed.

### 2.3. Assays

All biomarkers were analysed through commercially available kits. PTX3 was quantified in plasma samples through an enzyme-linked immunosorbent assay (ELISA) kit (Human Pentraxin 3/TSG-14 Quantikine ELISA Kit, R&D Systems, Minnesota, USA). Three classical inflammatory biomarkers were evaluated in serum samples: high-sensitivity (hs) CRP by immunoturbidimetry (Cardiac C-Reactive Protein (Latex) High Sensitive assay, Roche Diagnostics, Basel, Switzerland), IL-6, and TNF-*α* (Human IL-6 Quantikine HS and Human TNF-alpha Quantikine HS, R&D Systems). Lipid profile, including total cholesterol, triglycerides, high-density lipoprotein cholesterol (HDL-C), and low-density lipoprotein cholesterol (LDL-C), was performed using laboratorial routine procedures (Cobas Integra 400 Plus autoanalyser; Roche Diagnostics, Basel, Switzerland); plasma oxidized LDL (oxLDL) and serum adiponectin, and leptin levels were determined through ELISA kits (Oxidized LDL ELISA Kit, Mercodia AB, Uppsala, Sweden; Human Total Adiponectin/Acrp30 Quantikine ELISA Kit and Human Leptin Quantikine ELISA Kit, R&D Systems). Levels of plasma *N*-terminal pro-B-type natriuretic peptide (NT-proBNP) and serum tissue inhibitor of metalloproteinase-1 (TIMP-1) (Human proBNP and Human TIMP1 ELISA kits, Abcam, Cambridge, UK) were assessed as cardiac and renal fibrosis markers, respectively.

### 2.4. Statistical Analysis

Data were analysed using the IBM SPSS software, version 24 for Windows 10 (IBM, New York, USA). Data distribution was evaluated by the Shapiro-Wilk test. Results are presented as mean ± standard deviation or median (interquartile range) for parametrically or nonparametrically distributed variables, respectively. For categorical variables, differences between groups at baseline were analysed using the chi-squared test. For continuous variables, differences between groups were evaluated using Student's unpaired *t*-test or Mann-Whitney *U* test, depending on their distribution. Since the median age of the control group is significantly lower than that of the patient group, adjustment of group analysis for this confounding factor was performed using analysis of covariance, followed by Bonferroni correction. Since it is well documented that the adipose tissue secretion rate of leptin is significantly higher in women [27], an additional adjustment for gender distribution was performed for this adipokine. The strength of the correlations between variables was determined through the Spearman's rank correlation coefficient (*r*_S_). For analysis requiring normally distributed variables, nonparametric data were transformed as previously described [28]. To determine independent predictors of multiple variables in ESRD patients, a multiple regression analysis was performed using stepwise selection and independent variables were excluded from the predictive models when the collinearity index was over 15. Due to the redundant contribution of LDL-C and total cholesterol (*r*_S_ > 0.7) to the prediction models of each inflammatory marker, the latter variable was excluded from the regression analysis. Estimates of the all-cause mortality hazard ratio by an inflammatory marker in this patient cohort were determined by Cox regression analysis. A *p* < 0.05 value was considered statistically significant.

## 3. Results

Circulating levels of inflammatory biomarkers, lipid profile and adipokines, NT-proBNP, and TIMP-1 for the control group and ESRD patients are presented in Table 2. The concentration of PTX3 was 2.2-fold higher in ESRD patients, when compared to controls (*p* < 0.001). The other inflammatory biomarkers, CRP, IL-6, and TNF-*α*, as well as NT-proBNP and TIMP-1, were significantly increased in ESRD patients (*p* < 0.001). Regarding the lipid profile and adipokine levels, ESRD patients presented significantly higher levels of triglycerides and adiponectin (*p* < 0.001) and lower levels of total cholesterol (*p* < 0.001), HDL-C (*p* = 0.013), LDL-C (*p* < 0.001), and oxLDL (*p* < 0.001). All these differences remained significant after adjustment for age, with exception of the NT-proBNP levels (*p* = 0.054). The serum leptin levels were significantly higher in ESRD patients, after adjustment for this confounding factor plus gender distribution (*p* = 0.008). We found similar oxLDL/LDL-C ratios for controls and ESRD patients (*p* = 0.940).

Table 3 depicts the correlations of PTX3 with the other inflammatory markers and cardiac and renal fibrosis markers. Positive and significant correlations were observed between PTX3 and all classical inflammatory biomarkers CRP, IL-6, and TNF-*α* (*p* < 0.05). With the exception of TNF-*α*, all inflammatory markers were positively associated with NT-proBNP and TIMP-1 (*p* < 0.01).

**Figure 1:**
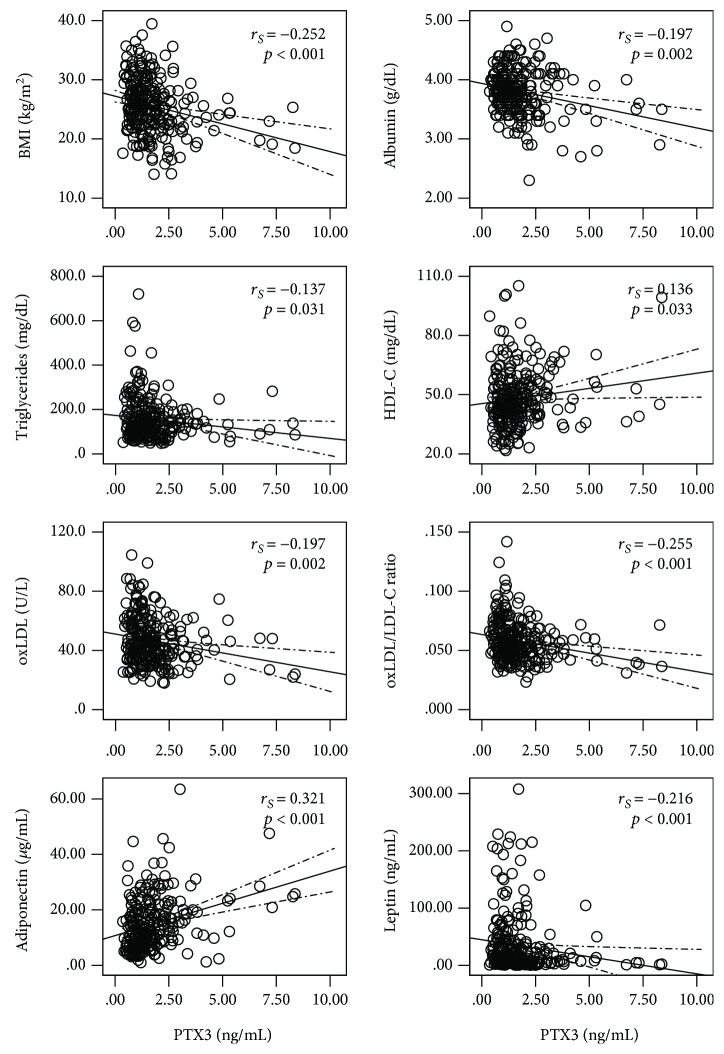
Correlation of PTX3 with the nutritional biomarkers and lipid profile in end-stage renal disease patients. *r*_S_: Spearman's rank correlation coefficient; PTX3: pentraxin 3; BMI: body mass index; HDL-C: high-density lipoprotein cholesterol; oxLDL: oxidized low-density lipoprotein; LDL-C: low-density lipoprotein cholesterol.

PTX3 levels showed, in ESRD patients, an inverse association with BMI and albumin levels; PTX3 was further negatively correlated with triglycerides, oxLDL concentration, oxLDL/LDL-C ratio, and leptin levels, while it positively correlated with HDL-C and adiponectin levels (*p* < 0.05) (Figure 1). A negative association of HDL-C with CRP (*r*_S_ = −0.229, *p* < 0.001), IL-6 (*r*_S_ = −0.156, *p* = 0.014), and TNF-*α* (*r*_S_ = −0.188, *p* = 0.003) was also observed. We also found significant correlations of albumin concentration with TNF-*α* (*r*_S_ = 0.136, *p* = 0.033) and IL-6 levels (*r*_S_ = −0.182, *p* = 0.004) and between BMI and TNF-*α* (*r*_S_ = 0.145, *p* = 0.023). Importantly, the observed correlations of PTX3 with triglycerides, oxLDL levels, oxLDL/LDL-C ratio, adiponectin, and leptin concentrations were not observed for any other inflammatory marker under study (data not shown).

As depicted in Table 4, the cardiac marker NT-proBNP appears as the best predictor of PTX3 levels in this patient cohort (*p* < 0.001), followed by the oxLDL/LDL-C ratio (*p* < 0.001) and TIMP-1 (*p* = 0.006). On the other hand, levels of CRP, IL-6, and TNF-*α* were mainly predicted by another inflammatory marker other than PTX3 (*p* < 0.001). Furthermore, PTX3 was the main inflammatory determinant of both cardiac and renal fibrosis markers (higher standardized beta value; lower *p* value) in these patients.

**Figure 2:**
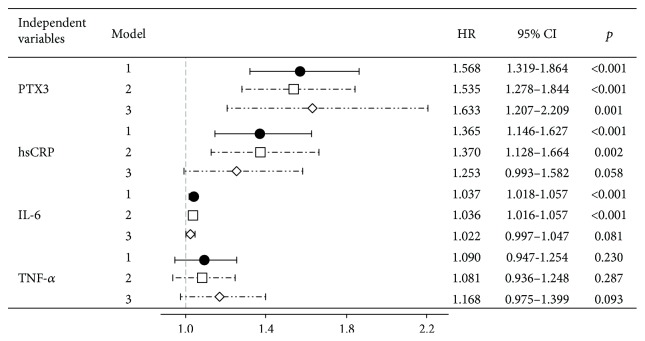
Univariate and multivariate Cox regression analysis of the inflammatory biomarkers for all-cause mortality in end-stage renal disease patients. Model 1 (filled circles): unadjusted hazard ratios. Model 2 (open squares): hazard ratios adjusted for age, dialysis vintage, and vascular access. Model 3 (open lozenges): hazard ratios adjusted for confounding factors, lipid profile, adipokines, and nutritional, cardiac, and renal fibrosis markers. HR: hazard ratio; CI: confidence interval; PTX3: pentraxin 3; hsCRP: high-sensitivity C-reactive protein; IL-6: interleukin-6; TNF-*α*: tumor necrosis factor-alpha.

Throughout the one-year clinical follow-up, there was a total of 26 deceased patients (10.6%). The hazard ratio (HR) for each inflammatory biomarker in the study over this follow-up period is presented in Figure 2. For the unadjusted model, PTX3, CRP, and IL-6, but not TNF-*α*, were determined as independent predictors of all-cause mortality in ESRD patients on dialysis (*p* < 0.001). When adjusting the model for basic confounding factors in dialysis (age, dialysis vintage, and vascular access), PTX3 appeared as the most sensitive predictor of mortality in this cohort (higher HR than IL-6 and CRP), showing a HR of 1.535 (1.278–1.844), meaning that an increment of 1 ng/mL of circulating PTX3 increases the risk of death by 53.5% (*p* < 0.001). This association of all-cause mortality with the PTX3 levels is significantly strong even after adjustment of a HR for the confounding factors plus all other risk factors in the study, including nutritional, cardiac, and renal fibrosis markers, lipid profile, and adipokines (HR = 1.633 (1.207–2.209), *p* = 0.001), which was not observed for any other inflammatory marker.

## 4. Discussion

Persistent low-grade inflammation has a worryingly high prevalence in CKD, being particularly enhanced in dialysis patients [1]. It has also been linked to the development and/or progression of several clinical comorbid conditions, including CVD, atherosclerosis, and malnutrition [8, 29]. Thus, dialysis patients present increased circulating levels of acute-phase reactants, such as CRP [30], and proinflammatory cytokines, like IL-6 [31] and TNF-*α* [32]. Accordingly, we found that levels of CRP, IL-6, and TNF-*α*, as well as PTX3, were significantly higher in the present cohort of ESRD patients on dialysis, when compared to healthy individuals (Table 2). This comes in line with other studies on PTX3 in CKD patients, either on dialysis or not [21, 33, 34]. Moreover, the potential of PTX3 as a biomarker of the inflammatory status in dialysis patients was further supported by the positive association between PTX3 concentration and all three classical inflammatory biomarkers in the present study (Table 3).

Alongside systemic inflammation, patients undergoing dialysis present a high prevalence of protein-energy malnutrition and increased susceptibility to develop atherosclerosis. This combination, termed “malnutrition-inflammation-atherosclerosis syndrome”, is commonly observed in CKD patients, suggesting a close cross talk between these three comorbid conditions [35].

Evaluation of malnutrition in CKD is a complex matter, and it is unlikely that a sole parameter will adequately phenotype this comorbidity. Serum albumin has been shown to be a limited predictor of malnutrition in this population [36, 37]. Nevertheless, measuring circulating albumin levels remains a readily available and a rather inexpensive assessment in Dialysis Clinics. Furthermore, low serum albumin levels, together with low BMI, are surrogate markers of protein-energy wasting syndrome in CKD [38, 39] and may therefore provide an overall estimation of the nutritional status of dialysis patients.

An inverse correlation between PTX3 levels and BMI, fat BMI, and serum albumin was described by Miyamoto et al. [40] in two independent cohorts of CKD patients; it was also shown that PTX3 is a better predictor of fat BMI than CRP in both ESRD patients on maintenance haemodialysis and in stage 5 CKD patients referred to start dialysis. Few studies have shown that hypoalbuminemia strongly associates with increased CRP and/or IL-6 levels in CKD patients [36, 41, 42], while data on the association of BMI with inflammation within these clinical settings is still contradictory [42–44]. In our dialysis cohort, we found that PTX3 levels correlated strongly and negatively with both BMI and serum albumin (Figure 1) and that this inverse association with BMI was unique of PTX3 and thus not observed with the classical inflammatory biomarkers. Wasting diseases, as CKD, especially ESRD, may disturb adipose tissue function and, therefore, the regulation of adipokines secretion. It was reported that nonoverweight patients undergoing haemodialysis presented lower leptin levels and higher adiponectin, as well as higher IL-6 and TNF-*α* concentrations, than overweight patients [45], and it has been suggested that adiponectin secretion may underlie a counteracting attempt against inflammatory damage in this population [46]. Taken together, these findings reinforce the idea of a close relationship between nutritional and inflammatory statuses in ESRD. In accordance with this data and with the negative association of BMI with PTX3, we found for PTX3 an inverse correlation with leptin and a positive correlation with adiponectin. Considering that PTX3 is expressed in different tissues, including in the adipose tissue [13], this inflammatory biomarker seems to be more sensitive to inflammation-related malnutrition than CRP, IL-6, and TNF-*α* in this population.

Both systemic inflammation and the changes in the lipid profile of ESRD patients are known to play a key role in the pathophysiology of atherosclerosis [47, 48]. For instance, high CRP levels have been linked with the progression of atherosclerosis in uraemia [49] and evidences suggest that CRP may play a direct role in atherogenesis [50]. Accordingly, we found that the CRP levels in ESRD patients were strongly and inversely associated with HDL-C (*r*_S_ = −0.229, *p* < 0.001), a lipoprotein known for its antiatherogenic effect, which appears to be dysfunctional in ESRD patients [51], supporting a possible involvement of CRP in atherogenesis in these patients.

PTX3 was shown to be strongly expressed in human atherosclerotic plaque [18]. However, the specific effects of this acute-phase protein in atherosclerosis remain a controversial matter [52], and even opposite effects in different stages of the disease have been hypothesized [53]. In fact, PTX3 appears to activate different pathways with contrasting consequences towards atherogenesis [24]. Increasing data suggest that the effects of PTX3 in atherogenesis, and consequently CVD, may depend on the clinical condition under study. For example, a proatherogenic role has been suggested for PTX3 in metabolic diseases [54, 55]. In contrast, the work of Nakamura et al. [56] in coronary artery disease patients showed that PTX3 correlates to body fat and lipid profile in a comparable way to adiponectin—an atheroprotective adipokine— and opposite to CRP. Furthermore, *in vivo* studies showed that PTX3 is cardioprotective in acute myocardial infarction [57], while deficiency in this pentraxin promotes both vascular inflammation and atherosclerosis in mice [15].

In our cohort of ESRD patients on dialysis, PTX3 displays an inverse association with an atherogenic lipid profile, supporting atheroprotective effects of PTX3 in these clinical settings. Unlike CRP, the PTX3 levels were positively correlated with the HDL-C levels (Figure 1). In fact, PTX3 is released by the major cell types involved in atherosclerotic lesions, namely, vascular endothelial cells and smooth muscle cells, macrophages, and neutrophils, and its production is induced by anti-inflammatory and atheroprotective signals, such as IL-10 and HDL-C itself [12]. Of note, recent *in vitro* studies provided evidence supporting an anti-inflammatory and, thus, a protective role for PTX3, by regulating macrophage activity to resolve inflammation [58, 59] rather than potentiating it, as occurs with CRP [60]. These contrasting effects of PTX3 and CRP on inflammation may underlie their putative opposing atherogenic effects in ESRD patients undergoing dialysis.

The adipocyte-derived proteins, adiponectin and leptin, with opposite inflammatory and atherogenic effects, are involved in protein-energy malnutrition and in the development of atherosclerotic CVD in CKD patients [61]. Furthermore, according to our recent findings, the higher adiponectin levels observed in ESRD patients seem to favour HDL modifications towards an antiatherogenic profile, from smaller to larger subfractions, as well as antioxidant protection for LDL particles [62]. In accordance to these findings and with the work of Miyamoto et al. [40], we found a positive correlation of PTX3 with adiponectin and a negative correlation with leptin in ESRD patients (Figure 1), further sustaining the antiatherogenic role for PTX3 in these patients. Importantly, we found that the correlations between these adipokines and the studied inflammatory markers were only statistically significant with PTX3, suggesting a closer relationship of adipocyte disturbances with PTX3 rather than with CRP, IL-6, and TNF-*α*.

PTX3 levels were also negatively associated with both oxLDL concentration and oxLDL/LDL-C ratio (Figure 1), and regression analysis showed that the latter one is an independent negative determinant of the PTX3 levels in dialysis patients (Table 4). In contrast, Liu et al. [63] showed that PTX3 affects lipid accumulation in human macrophages, promoting the uptake of oxLDL by macrophages, supporting a proatherogenic mechanism for PTX3. Further studies are required to confirm the actual outcome of the balance between the pro- and antiatherogenic mechanisms of PTX3 in the development and/or progression of atherosclerosis in dialysis patients.

Our data analysis also showed a strong and positive association between the PTX3 levels and NT-proBNP in patients undergoing dialysis, at a greater extent than CRP (Table 3), while the linear regression analysis showed that the NT-proBNP levels are also independent predictors of PTX3 concentration in this cohort, and vice versa (Table 4). NT-proBNP is a well-known marker of CV risk in CKD patients [64], and these results come in line with the predictive value of PTX3 in cardiovascular outcomes previously described in nondialysis CKD patients [21, 34]. Nevertheless, as for atherosclerosis, there is still some controversial views about the effect of PTX3 and its contribution to CVD [24] and further investigation is required to assess its role, specifically in ESRD settings.

TIMP-1 is the major endogenous regulator of matrix metalloproteinase-9 (MMP-9) which, through multiple signalling pathways, is linked to the occurrence and progression of CKD [65]. A recent study showed that long-term low-grade inflammation, with sustained increased PTX3 levels, may underlie renal fibrosis in ESRD due to a MMP-9/TIMP-1 imbalance [66]. Moreover, *in vitro* studies showed that PTX3 appears to contribute to renal fibrosis by promoting differentiation of monocytes into fibrocytes and upregulating epithelial-to-mesenchymal transition [67, 68]. On the other hand, PTX3 was shown to be innately involved in tissue remodelling and repair under an acidic environment [69], occurring under conditions such as hypoxia, which may support a protective role of PTX3 during renal damage. Nevertheless, the strong correlation found between the PTX3 levels and circulating TIMP-1 in our cohort of dialysis patients (Table 3), as well as the regression data from the predictive analysis (Table 4), supports a possible association between this modulator and renal fibrosis in ESRD patients. However, it remains unclear which is the specific effect or balance of effects of PTX3 in the development of this comorbid condition in dialysis patients.

Despite the increase in survival rates in the past decade, ESRD patients undergoing dialysis are still a very susceptible population, with an unacceptably high mortality prevalence worldwide [70]. Therefore, a more precise assessment of risk is essential for an accurate prognosis and treatment of ESRD patients on dialysis. Previous studies showed that high levels of inflammatory biomarkers, like CRP, IL-6, and PTX3, but not TNF-*α*, independently predict mortality in dialysis patients [22, 23, 71, 72], which is in accordance with our results from the univariate Cox regression analysis (*p* < 0.001 for the HR of CRP, IL-6, and PTX3). However, we first describe that PTX3 remains the most sensitive inflammatory predictor of all-cause mortality after adjustment for the main confounding factors in dialysis (HR = 1.535, *p* < 0.001) and preserves its predictive potential even after adjustment for all markers under study (HR = 1.633, *p* = 0.001) (Figure 2).

Considering that PTX3 is rapidly produced and released by damaged tissue cells, unlike CRP, which is produced mainly in the liver and reflects a systemic inflammatory response [12], the long pentraxin reflects the local inflammatory status of different tissues. Additionally, due to its local and rapid release, the circulating PTX3 levels could suffer alterations prior to a detectable increase in serum CRP concentration, as described in other clinical conditions [26, 46, 67] and even during a dialysis session [54]. This finding would be of a great value for an earlier clinical diagnosis and treatment of inflammation in dialysis patients.

In view of the multiplicity of comorbidities in advanced CKD and ESRD patients on dialysis, PTX3 appears to be a broader marker for multiple risk factors than the standard inflammatory marker CRP. Furthermore, PTX3 may be useful in a multibiomarker approach potentially providing more accurate clinical information than the analysis of an individual marker.

Some limitations should be taken into account when evaluating the relevance of the present findings. For instance, the complexity of comorbid conditions and the multiple drug therapies (statins, diuretics, antihypertensives, erythropoiesis-stimulating agents, iron, etc.) represent a major limitation when interpreting data from ESRD patients; notwithstanding the fairly large cohort, the study is still limited by the sample size with regard to mortality analysis, partially due to the narrow follow-up period analysed; a single blood collection at a certain time point may not accurately reflect the natural course of the process in the study. Nevertheless, the present study comprises a representative sample of the Portuguese ESRD population; the impact of potential bias in data obtained was reduced through adjustment of analysis for common confounding factors, and the range of values observed for each parameter in the present study is similar to that of cohort studies on different ESRD populations, thus supporting the external validity of the present findings.

## 5. Conclusions

The present study provides evidence supporting the association of PTX3 with multiple risk factors associated with ESRD patients undergoing dialysis, being a plausible clinical target to counteract the high morbidity and mortality associated with advanced CKD. Data further upholds the usefulness of standardizing a high-sensitivity method for PTX3 measurement as a wide-ranging clinical biomarker, with potential to assess inflammatory status, malnutrition, CVD, and renal fibrosis and to predict all-cause mortality in dialysis patients with a greater accuracy than the conventional inflammatory biomarkers.

## Figures and Tables

**Table 1:** Demographic, biochemical, and dialysis-related data for controls and end-stage renal disease patients.

	Controls (*n* = 22)	ESRD patients (*n* = 246)	*p*
Gender, *n* (%)			
Male	8 (36.4)	134 (54.5)	0.121
Female	14 (63.6)	112 (45.5)	
Age (years)	56.9 [52.3–59.8]	71.0 [59.7–79.5]	**<0.001**
BMI (kg/m^2^)	24.3 ± 3.4	25.6 ± 4.7	0.215
Aetiology of CKD, *n* (%)			
Diabetic nephropathy	—	87 (35.4)	—
Hypertensive nephrosclerosis		34 (13.8)	
Polycystic kidney disease		17 (6.9)	
Chronic glomerulonephritis		18 (7.3)	
Other or undetermined		90 (36.6)	
Dialysis vintage (years)		3.87 [1.79–7.48]	
Dialysis therapy, *n* (%)			
Haemodialysis	—	30 (12.2)	—
Online haemodiafiltration		216 (87.8)	
Vascular access, *n* (%)			
Arteriovenous fistula	—	199 (80.9)	—
Arteriovenous graft		12 (4.9)	
Central venous catheter		35 (14.2)	
Biochemical and dialysis markers			
Sodium (mEq/L)	—	137 [135-139]	—
Potassium (mEq/L)		5.16 ± 0.74	
Phosphorus (mg/dL)		4.14 [3.31–4.99]	
Calcium (mg/dL)		8.94 ± 0.55	
Calcium phosphorus product		36.74 [29.75–44.96]	
Albumin (g/dL)		3.8 [3.6–4.1]	
URR (%)		79.0 [75.8–83.0]	
eKt/V		1.62 ± 0.28	
Ultrafiltration volume (L)		2.3 [1.7–2.9]	

Data are presented as mean ± standard deviation or as median (interquartile range). ESRD: end-stage renal disease; BMI: body mass index; URR: urea reduction ratio.

**Table 2:** Circulating levels of inflammatory markers, lipid profile, adipokines, and cardiac and renal fibrosis biomarkers in controls and end-stage renal disease patients.

	Controls (*n* = 22)	ESRD patients (*n* = 246)	*p*
Inflammatory markers			
PTX3 (ng/mL)	0.62 [0.50–0.74]	1.40 [0.96–2.06]	**<0.001**
hsCRP (mg/dL)	0.10 [0.04–0.20]	0.36 [0.18–0.77]	**<0.001**
IL-6 (pg/mL)	1.04 [0.68–1.54]	4.09 [2.64–7.47]	**<0.001**
TNF-*α* (pg/mL)	0.83 [0.71–1.09]	3.31 [2.65–4.45]	**<0.001**
Lipid profile and adipokines			
Total cholesterol (mg/dL)	204.0 [188.2–221.2]	159.5 [133.8–188.0]	**<0.001**
Triglycerides (mg/dL)	90.0 [71.2–107.5]	133.0 [97.5–183.2]	**<0.001**
HDL-C (mg/dL)	52.6 [44.7–65.4]	45.2 [38.9–55.1]	**0.013**
LDL-C (mg/dL)	122.2 [104.4–137.9]	79.6 [64.5–104.5]	**<0.001**
oxLDL (U/L)	67.5 [55.4–73.5]	44.4 [34.1–56.4]	**<0.001**
oxLDL/LDL-C ratio	0.055 [0.049–0.064]	0.054 [0.046–0.067]	0.940
Adiponectin (*μ*g/mL)	5.75 [3.02–8.35]	12.32 [7.86–20.00]	**<0.001**
Leptin (ng/mL)	9.34 [4.94–21.61]	13.77 [4.82–38.88]	0.180^b^
Cardiac marker			
NT-proBNP (ng/mL)	N.D. [N.D.–0.25]^a^	13.57 [8.21–25.23]	**<0.001** ^c^
Renal fibrosis marker			
TIMP-1 (ng/mL)	243.0 [212.2–325.0]	537.0 [467.8–627.2]	**<0.001**

Data are presented as median (interquartile range). ^a^Levels of NT-proBNP were below the detection limit of the technique for a total of 12 control samples. ^b^*p* = 0.008 and ^c^*p* = 0.054 after adjustment for confounding factors. ESRD: end-stage renal disease; PTX3: pentraxin 3; hsCRP: high-sensitivity C-reactive protein; IL-6: interleukin-6; TNF-*α*: tumor necrosis factor-alpha; HDL-C: high-density lipoprotein cholesterol; LDL-C: low-density lipoprotein cholesterol; oxLDL: oxidized LDL; NT-proBNP: *N*-terminal pro-B-type natriuretic peptide; TIMP-1: tissue inhibitor of metalloproteinase-1.

**Table 3:** Correlations between PTX3 and other inflammatory markers, with each other, and cardiac and renal fibrosis markers, in end-stage renal disease patients.

		PTX3	hsCRP	IL-6	TNF-*α*
Inflammatory markers					
hsCRP	*r*_S_	0.157	—	—	—
	*p*	**0.013**			
IL-6	*r*_S_	0.197	0.583	—	—
	*p*	**0.002**	**<0.001**		
TNF-*α*	*r*_S_	0.142	0.259	0.277	—
	*p*	**0.026**	**<0.001**	**<0.001**	
Cardiac marker					
NT-proBNP	*r*_S_	**0.344**	**0.191**	**0.281**	0.120
	*p*	**<0.001**	**0.003**	**<0.001**	0.061
Tubular injury marker					
TIMP-1	*r*_S_	**0.281**	**0.270**	**0.268**	0.105
	*p*	**<0.001**	**<0.001**	**<0.001**	0.101

*r*_S_: Spearman's rank correlation coefficient; PTX3: pentraxin 3; hsCRP: high-sensitivity C-reactive protein; IL-6: interleukin-6; TNF-*α*: tumor necrosis factor-alpha; NT-proBNP: *N*-terminal pro-B-type natriuretic peptide; TIMP-1: tissue inhibitor of metalloproteinase-1.

**Table 4:** Multiple linear regression model of prediction of each inflammatory biomarker under study, considering all other evaluated biomarkers, and NT-proBNP and TIMP-1, considering all inflammatory markers in end-stage renal disease patients.

Dependent variable	Model	Unstandardized coefficients		Standardized coefficients	*t*	*p*
		*B*	Std. error	Beta		
PTX3	(Constant)	1.721	0.382		4.504	<0.001
	NT-proBNP	0.008	0.002	0.244	3.960	**<0.001**
	oxLDL/LDL-C ratio	-14.772	4.327	-0.202	-3.414	**<0.001**
	TIMP-1	0.001	0.000	0.173	2.787	**0.006**

hsCRP	(Constant)	-0.117	0.324		-0.361	0.718
	IL-6	0.071	0.007	0.547	10.505	**<0.001**
	HDL-C	-0.011	0.004	-0.137	-2.718	**0.007**
	LDL-C	0.005	0.002	0.127	2.543	**0.012**
	TIMP-1	0.001	0.000	0.127	2.462	**0.015**

IL-6	(Constant)	3.206	0.532		6.023	<0.001
	hsCRP	4.588	0.394	0.600	11.657	**<0.001**

TNF-*α*	(Constant)	3.516	0.171		20.592	<0.001
	IL-6	0.069	0.016	0.271	4.383	**<0.001**

NT-proBNP	(Constant)	-1.079	4.232		-0.255	0.799
	PTX3	9.041	1.950	0.282	4.635	**<0.001**
	IL-6	0.882	0.275	0.195	3.209	**0.002**

TIMP-1	(Constant)	491.588	18.095		27.167	<0.001
	PTX3	32.583	8.419	0.237	3.870	**<0.001**
	hsCRP	32.720	9.074	0.221	3.606	**<0.001**

PTX3: pentraxin 3; NT-proBNP: *N*-terminal-pro-B-type natriuretic peptide; oxLDL/LDL-C: oxidized low-density lipoprotein/low-density lipoprotein cholesterol; TIMP-1: tissue inhibitor of metalloproteinase-1; hsCRP: high-sensitivity C-reactive protein; IL-6: interleukin-6; HDL-C: high-density lipoprotein cholesterol; LDL-C: low-density lipoprotein cholesterol; TNF-*α*: tumor necrosis factor-*α*.

## Data Availability

The data used to support the findings of this study are included within the article.
